# Mechanical Thrombectomy for Treatment of Acute Cerebral Infarction due to Distal Medium Vessel Occlusions: A Retrospective Cohort Study

**DOI:** 10.1002/brb3.70119

**Published:** 2024-11-07

**Authors:** Lihong Zhang, Fanfan Su, Jianhui Zhang, Jia Xu, Manhong Zhao, Di Li, Lin Yin

**Affiliations:** ^1^ Department of Neurointervention and Neurocritical Care Dalian Central Hospital Affiliated to Dalian University of Technology Dalian China; ^2^ Department of Neurology The Second Hospital of Dalian Medical University Shahekou District Liaoning China; ^3^ Department of Neurology 967 Hospital of PLA Joint Logistic Support Force Dalian City Liaoning Province China

**Keywords:** acute cerebral infarction, distal medium vessel occlusions, efficacy, mechanical thrombectomy, prognosis, safety

## Abstract

**Background:**

Mechanical thrombectomy (MT) is standard of care for acute cerebral infarction (ACI) due to large vessel occlusions. However, its clinical efficacy in patients with ACI due to distal medium vessel occlusions (DMVOs) remains unclear. This study evaluates the efficacy and safety of MT in patients with ACI due to DMVOs.

**Methods:**

Totally, 306 patients with ACI at a very early stage were assigned into DMVOs‐MT, M1‐MT, and DMVOs‐intravenous thrombolysis (IVT) groups. These groups were compared regarding baseline data, recanalization rate, location of vessel occlusions, number of thrombectomy, first‐pass recanalization, mRS scores, NIHSS scores, 90‐day mRS scores, incidence of adverse events, and mortality. Risk factors for poor prognosis of patients with DMVOs following MT were analyzed.

**Results:**

DMVOs‐MT and M1‐MT groups showed comparable first‐pass recanalization rates, recanalization rates, and NIHSS score reduction ratios, with marked differences in location of vessel occlusions. Versus DMVOs‐IVT, DMVOs‐MT had increased differences between pre‐ and post‐treatment NIHSS scores and between pre‐treatment NIHSS scores and NIHSS scores at discharge and elevated NIHSS reduction ratios. The poor prognosis rate of DMVOs‐MT group was insignificantly different from that of M1‐MT group but lower than that of DMVOs‐IVT group. Adverse events and mortality incidences were comparable among the three groups. Diabetes, first‐pass recanalization, and pre‐treatment NIHSS scores were independent risk factors for poor prognosis in DMVO patients after MT.

**Conclusion:**

MT is as effective and safe in patients with DMVOs as in patients with M1 occlusions. In patients with DMVOs, MT has higher efficacy and safety than IVT.

## Graphical abstract

The efficacy of MT in patients with cerebral infarction due to DMVOs is comparable to that of MT in patients with cerebral infarction due to MI segment occlusions and is superior to that of IVT with alteplase in patients with DMVOs, with higher safety.

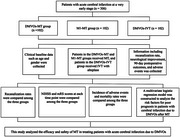

## 1 Introduction

Ischemic stroke constitutes the majority of stroke cases, which is the leading cause of adult disability and mortality worldwide (Ajoolabady et al. 2021; Moretti, Ferrari, and Villa 2015). Cerebral infarction is among the main causes of ischemic stroke (Zhao et al. 2022). As a prevalent cerebrovascular disease, cerebral infarction is characterized by behavioral, cognitive, and language impairment, seriously threatening the health and quality of life of patients (Xu et al. 2023). It is estimated that 25%–40% of acute cerebral infarction is due to distal medium vessel occlusions (DMVOs) attributed to primary thromboemboli and consequences arising from endovascular thrombectomy (EVT) performed for proximal large vessel occlusions (Saver et al. 2020). DMVOs are defined as thromboembolic occlusions in the anterior cerebral artery, M2–M4 segments of the middle, posterior inferior, posterior, anterior inferior, and superior cerebellar arteries (Kobeissi et al. 2023). Despite less severe clinical symptoms than proximal occlusions, DMVOs, particularly those in the dominant cerebral hemisphere, can cause significant morbidity and disability (Munich, Vakharia, and Levy 2019; Toh et al. 2023). Although intravenous thrombolysis (IVT) is the preferred treatment option for DMVOs, the application of this option is limited due to its insufficient efficacy when used alone (Burel et al. 2023).

Mechanical thrombectomy (MT), a minimally invasive procedure, is recommended in acute cerebral infarction patients with large vessel occlusions when treatment with IVT is contraindicated (Derex and Cho 2017; Xiong, Wakhloo, and Fisher 2022). Recently, MT is well established as a highly effective and safe treatment modality for acute cerebral infarction patients with DMVOs by improving recanalization rates and reducing deleterious consequences (Piscopo, Zanaty, and Dlouhy 2023; Leung et al. 2018; Pouget et al. 2023). According to previous retrospective studies, the efficacy and safety of MT in the treatment of distal arterial occlusion strokes are comparable to those in the treatment of proximal arterial occlusion strokes (Sweid et al. 2019; Nogueira et al. 2021). Daniel et al. found comparable clinical outcomes and safety of MT in stroke patients with M2 occlusions to those in patients with M1 occlusions (Weiss et al. 2022). Therefore, this retrospective cohort study further analyzed the efficacy and safety of MT in treating patients with acute cerebral infarction due to DMVOs and evaluated risk factors for poor prognosis in patients following MT, thus providing a certain reference basis for the large‐scale application of MT in the treatment of cerebral infarction due to DMVOs.

## 2 Materials and Methods

### 2.1 Study Subjects

This retrospective cohort study included 306 patients with acute cerebral infarction at a very early stage admitted at comprehensive stroke centers (Dalian, China; the Second Hospital affiliated to Dalian Medical University, Dalian Municipal Central Hospital affiliated to Dalian University of Technology, and the 967^th^ Hospital of Chinese People's Liberation Army Joint Logistic Support Force) from December 1, 2016 to November 20, 2022. These patients were allocated into three groups (*n* = 102 per group) based upon the treatment they received: DMVOs‐MT (patients with acute cerebral infarction underwent MT for DMVOs), M1‐MT (patients underwent MT for M1 segment occlusions), and DMVOs‐IVT (patients underwent IVT with alteplase for DMVOs). The ethics committee of The Second Hospital of Dalian Medical University approved this study, which was performed in strict accordance with the *Declaration of Helsinki*.

### 2.2 Inclusion and Exclusion Criteria

Inclusion criteria for participants were as follows: (1) participants with acute cerebral infarction at a very early stage who met the diagnostic criteria for acute cerebral infarction in the guidelines for the management of patients with acute ischemic stroke published by the American Heart Association Stroke Council (2018) (Powers et al. 2018); (2) participants presenting with occlusions in the M1 segment of middle cerebral artery, A2/A3 segments of anterior cerebral artery, and M2/M3 segments of middle cerebral artery, which were confirmed by magnetic resonance angiography, head and neck CT angiography, or digital subtraction angiography (DSA); (3) participants with complete clinical information; (4) patients undergoing MT or IVT with alteplase using approved equipment (suction catheter or stent retriever). Exclusion criteria were as follows: (1) patients with missing data; (2) patients combined with other malignant diseases; (3) patients with severe heart, lung, and renal dysfunction(s).

### 2.3 Treatment Procedures

For MT, patients were placed in a supine position and general anesthesia with endotracheal intubation was induced. The right femoral artery was punctured with the modified Seldinger method, where an 8F arterial catheter (Cordis Corporation, Miami, FL, USA) was inserted and heparinized normal saline was infused intravenously. Then, an 8F guiding catheter (Cordis Corporation) or Neuron Max 6F long sheath (Penumbra Inc., Alameda, CA, USA) was placed in the appropriate position of the internal carotid artery on the lesion side through the 125 mm long single‐bend catheter (Cordis Corporation), followed by DSA at anteroposterior, lateral, and ipsilateral 45° oblique. If necessary, 3D‐DSA was performed to find the best operating position.

Stent thrombectomy: the tip of the Navien 058 intermediate catheter (ev3 Inc., Irvine, CA, USA) was placed at the C4 segment under the support of the 0.014‐inch microguidewire (Synchro‐14; 200 cm in length, Boston Scientific, Marlborough, MA, USA) and the Rebar‐18 microcatheter (Covidien, Irvine, CA, USA). After the microguidewire passed through the occluded segment, the microcatheter end was introduced to the distal end of the M2 segment, and the microguidewire was withdrawn. “Smoking” in the microcatheter confirmed the location in the lumen of the blood vessel. The solitaire AB stent (4 mm × 20 mm; ev3 Inc.) was released after accurate positioning and allowed to stand for a 5–m duration. The Navien catheter was moved as far as possible to the proximal end of the occluded segment. SWIM technology was applied to slowly withdraw the stent, microcatheter, and Navien catheter under negative pressure suction. DSA was reviewed immediately after thrombectomy, and successful recanalization was confirmed in the presence of the modified thrombolysis in cerebral infarction (mTICI) score ≥ 2b. As such, the surgery was completed. If the vessel recanalization failed, MT with stent was performed again according to the method mentioned above. Such surgery could not be performed more than three times to reduce the risk of vascular damage and intracranial hemorrhage.

Direct suction thrombectomy: an appropriate suction catheter such as 5F Sofia (MicroVention, Aliso Viejo, CA, USA), 4MAX, or 3MAX (Penumbra Inc.) was selected based on the preoperative DSA results. With the support of the microguidewire and microcatheter, the suction catheter was guided to the occluded segment to reach the proximal end of the thrombus. Next, negative pressure suction was conducted with a 50 mL syringe for 90 s, and the catheter was slowly removed without bending throughout the process under negative pressure. DSA was reviewed immediately after thrombectomy, and successful recanalization was confirmed in the presence of the mTICI score ≥ 2b. As such, the surgery was completed. If the mTICI score was < 2b after three times of suction, stent thrombectomy was performed.

IVT with alteplase: alteplase (Boehringer Ingelheim Pharmaceutical Inc., Ingelheim, Germany; National Medical Products Administration [NMPA] Approval No. SJ20160054; 20 mG; the total dose of 0.9 mG/kg) was intravenously injected into patients within 4.5 h of symptom onset. For alteplase injection, 10% of the total dose was injected intravenously within 1 m initially, and the remaining 90% was mixed with 100 mL of 0.9% sodium chloride (Sichuan Kelun Pharmaceutical Co., Ltd., Chengdu, China; NMPA Approval No. H20056626; 100 mL:5 g) for intravenous infusion within 1 h. During clinical treatment, the blood pressure of patients was stabilized at 100–180 mmHg (1 mmHg = 0.133 kPa). After thrombolytic treatment, patients were given oral enteric‐coated aspirin tablets (CSPC Ouyi Pharmaceutical Co., Ltd., Shijiazhuang, Hebei, China; NMPA Approval No. H20153035; 100 mG) once daily.

### 2.4 Evaluation of Efficacy and Safety

Recanalization of patients was evaluated according to the mTICI scoring system (Monteiro et al. 2024). The mTICI scoring system was graded as follows: grade 0, no perfusion; grade 1, perfusion of only a small amount of blood through the occluded segment, with little or no perfusion; grade 2a, partial perfusion of forward blood flow < 50% downstream of the ischemic area; grade 2b, partial perfusion of forward blood flow ≥ 50% downstream of the ischemic area; and grade 3, an ischemic area downstream of complete perfusion of forward blood flow. Successful recanalization was defined as a mTICI grade ≥ 2b.

Neurological function of patients was assessed before surgery, at 24 h after surgery, and at discharge with the National Institutes of Health Stroke Scale (NIHSS).

The outcomes were assessed post 90 days of surgery with the modified Rankin Scale (mRS) (van Swieten et al. 1988). An mRS score of ≤ 2 indicated favorable prognosis, and an mRS score of ≥ 3 represented poor prognosis.

Adverse events were recorded, including perforation, dissection, subarachnoid hemorrhage, and symptomatic intracranial hemorrhage. Intracranial hemorrhage was suggested by head CT within 72 h postoperatively, which could be classified as symptomatic and asymptomatic intracranial hemorrhages based on the aggravation of neurological deficits or not. This study concentrated on symptomatic intracranial hemorrhage, which was defined as cerebral hemorrhage according to neurological deterioration at the time of CT scanning and a clinical increase of at least 4 points in NIHSS scores and was attributable to parenchymal hematoma, intraventricular hemorrhage (von Kummer et al. 2015; Warach et al. 2023; Yu et al. 2023; Yan et al. 2019).

### 2.5 Clinical Data Acquisition

Electronic medical record system and original records were reviewed to collect clinical information of patients, including age, gender, smoking history, hypertension, diabetes, atrial fibrillation, Alberta Stroke Programme Early Computed Tomography Score (ASPECTS), Trial of ORG 10172 in Acute Stroke Treatment (TOAST) classification, thrombectomy plan, rescue plan, time from symptom onset to puncture, location of vessel occlusions, number of thrombectomy, first‐pass recanalization, pre‐and post‐treatment NIHSS scores, NIHSS scores at discharge, pre‐treatment mRS scores, mRS scores at discharge, 90‐day mRS scores, and incidence of adverse events.

### 2.6 Statistical Analysis

Data analysis and graphing were performed with SPSS 21.0 software (IBM Corp., Armonk, NY, USA) and GraphPad Prism 8.01 software (GraphPad Software Inc., San Diego, CA, USA). Measurement data were tested for normal distribution with the Shapiro‐Wilk test. Normally distributed measurement data were summarized as mean ± standard deviation and compared between two groups with the independent sample *t*‐test. Data with skewed distribution were presented as median (interquartile range) and compared between two groups with the Wilcoxon rank sum test. Categorical data, which were displayed as number of cases and percentages, were compared between two groups with the chi‐square test. Logistic regression analysis was employed to evaluate risk factors for poor prognosis in patients with acute cerebral infarction due to DMVOs following MT. Statistical tests were two tailed, and *p* < 0.05 indicated a significant difference.

## 3 Results

### 3.1 Comparison of Baseline Data Among the DMVOs‐MT, M1‐MT, and DMVOs‐IVT Groups

As depicted in Table 1, the DMVOs‐MT, M1‐MT, and DMVOs‐IVT groups showed no remarkable differences in terms of age, gender, smoking history, hypertension, diabetes, atrial fibrillation, ASPECTS, TOAST classification, pre‐treatment NIHSS scores, time from symptom onset to puncture, and mRS scores before cerebral infarction (*p* > 0.05). Compared with the M1‐MT group, the DMVOs‐MT group displayed shorter puncture‐to‐recanalization time and a higher percentage of cases taking rescue plans, with significant differences in location of vessel occlusions (all *p* < 0.05). No marked differences were found in the location of vessel occlusions between the DMVOs‐MT and DMVOs‐IVT groups (*p* > 0.05).

**TABLE 1:** Baseline data of patients in the DMVOs‐MT, M1‐MT, and DMVOs‐IVT groups (*n* = 102).

Items	M1‐MT	DMVOs‐IVT	DMVOs‐MT	*p_a_*	*p_b_*
Age (year)	65.37 ± 9.36	64.82 ± 8.71	65.67 ± 9.19	0.970	0.760
Gender [male, *n* (%)]	64 (62.75)	62 (60.78)	61 (59.80)	0.666	0.886
Smoking history [*n* (%)]	34 (33.33)	37 (36.27)	38 (37.25)	0.558	0.885
Hypertension [*n* (%)]	57 (55.88)	54 (52.94)	58 (56.86)	0.888	0.574
Diabetes [*n* (%)]	30 (29.41)	33 (32.35)	29 (28.43)	0.877	0.543
Aatrial fibrillation [*n* (%)]	43 (42.16)	47 (46.08)	44 (43.14)	0.888	0.673
Hyperlipidemia [*n* (%)]	46 (45.10)	43 (42.16)	48 (47.06)	0.779	0.481
ASPECTS	8.28 ± 1.70	8.29 ± 1.67	8.17 ± 1.69	0.887	0.868
TOAST classification	—	—	—	0.779	0.325
Large artery atherosclerosis [*n* (%)]	54 (52.94)	59 (57.84)	52 (50.98)		
Cardioembolic or other stroke subtypes [*n* (%)]	48 (47.06)	43 (42.16)	50 (49.02)		
Pre‐treatment NIHSS scores	15.21 ± 5.33	13.71 ± 4.56	13.80 ± 4.19	0.085	0.990
Time from symptom onset to puncture (min)	267.13 ± 122.26	—	278.02 ± 130.99	0.536	—
Time from puncture to recanalization [*n* (%)]	85.21 ± 42.37	—	68.99 ± 45.01	0.009	—
Pre‐treatment mRS scores	3.80 ± 0.81	3.93 ± 0.73	3.87 ± 0.71	0.521	0.562
Rescue plan [*n* (%)]	18 (17.65)	—	32 (31.37)	0.000	—
Thrombectomy plan		—		0.721	—
Suction [*n* (%)]	32 (31.37)	—	28 (27.45)		
Stent [*n* (%)]	46 (45.10)	—	52 (50.98)		
Suction plus stent [*n* (%)]	24 (23.53)	—	22 (21.57)		
Location of vessel occlusions [*n* (%)]				< 0.001	0.650
M1	102 (100.00)	—	—		
M2	—	93 (91.18)	89 (87.25)		
M3	—	8 (7.84)	10 (9.80)		
A1	—	1 (0.98)	2 (1.96)		
A2	—	0 (0.00)	1 (0.98)		

*Note*: Continuous variables with normal distribution were expressed as mean ± standard deviation and compared between two groups with the independent sample *t*‐test. Categorical data were displayed as number of cases and percentages [*n* (%)] and compared between two groups with the chi‐square test. *p_a_* indicates comparison between the DMVOs‐MT and M1‐MT groups. *p_b_* indicates comparison between the DMVOs‐MT and DMVOs‐IVT. *p* < 0.05 indicates a significant difference.

### 3.2 Efficacy in the DMVOs‐MT, M1‐MT, and DMVOs‐IVT Groups

The number of thrombectomy in the DMVOs‐MT group was significantly lower than that in the M1‐MT group, and the first‐pass recanalization rate and recanalization rate were equivalent between the two groups (*p* < 0.001, Table 2). Therefore, the efficacy of MT in patients with acute cerebral infarction due to DMVOs was comparable to that in patients with acute cerebral infarction due to M1 segment occlusions. The results in Table 3 exhibited higher recanalization rates in the DMVOs‐MT group than in the DMVOs‐IVT group (*p* < 0.001), illustrating that the efficacy of MT in patients with acute cerebral infarction due to DMVOs is superior to that of IVT in patients with acute cerebral infarction due to DMVOs.

**TABLE 2:** Comparison of efficacy between the DMVOs‐MT and M1‐MT groups (*n* = 102).

Items	M1‐MT	DMVOs‐MT	*p*
Number of thrombectomy	2.85 ± 0.55	2.45 ± 0.65	< 0.001
First‐pass recanalization rate (mTICI ≥ 2b)	45 (44.12)	52 (50.98)	0.454
Recanalization rate (mTICI ≥ 2b)	79 (77.45)	83 (81.37)	0.575

*Note*: Continuous variables with normal distribution were expressed as mean ± standard deviation and compared between two groups with the independent sample *t*‐test. Categorical data were displayed as number of cases and percentages [*n* (%)] and compared between two groups with the chi‐square test. *p* < 0.05 indicates a significant difference.

**TABLE 3:** Comparison of efficacy between the DMVOs‐MT and DMVOs‐IVT (*n* = 102).

Items	DMVOs‐IVT	DMVOs‐MT	*p*
Recanalization rate (mTICI ≥ 2b)	34 (33.33)	83 (81.37)	< 0.001

*Note*: Continuous variables with normal distribution were expressed as mean ± standard deviation and compared between two groups with the independent sample *t*‐test. Categorical data were displayed as number of cases and percentages [*n* (%)] and compared between two groups with the chi‐square test. *p* < 0.05 indicates a significant difference.

### 3.3 Analysis of Neurological Deficits in the DMVOs‐MT, M1‐MT, and DMVOs‐IVT Groups

No significant differences were observed in NIHSS scores before surgery among the DMVOs‐MT, M1‐MT, and DMVOs‐IVT groups (*p* > 0.05, Table 4). In comparison with the M1‐MT group, the DMVOs‐MT group exhibited lower differences between pre‐ and post‐treatment NIHSS scores and between pre‐treatment NIHSS scores and NIHSS scores at discharge (*p* < 0.05), with no differences found in the NIHSS score reduction ratio between the two groups (*p* > 0.05, Table 4). Conversely, differences between pre‐ and post‐treatment NIHSS scores and between pre‐treatment NIHSS scores and NIHSS scores at discharge, as well as the NIHSS score reduction ratio, were higher in the DMVOs‐MT group than in the DMVOs‐IVT group (all *p* < 0.05, Table 4). These results suggested that compared with IVT, MT had better performance in improving NIHSS scores in patients with acute cerebral infarction due to DMVOs. As observed in Table 5, mRS scores at discharge and 90‐day mRS scores in the DMVOs‐MT group were decreased relative to those in the remaining two groups. Additionally, the poor prognosis rate of the DMVOs‐MT group was similar to that of the M1‐MT group but lower than that of the DMVOs‐IVT group. These findings vindicate that MT in patients with acute cerebral infarction due to DMVOs leads to better neurological function improvement than IVT in such patients and results in equivalent poor prognosis rates to MT in patients with acute cerebral infarction due to M1 segment occlusions.

**TABLE 4:** NIHSS scores in the DMVOs‐MT, M1‐MT, and DMVOs‐IVT groups (*n* = 102).

Items	M1‐MT	DMVOs‐IVT	DMVOs‐MT	*p_a_*	*p_b_*
Pre‐treatment NIHSS scores	15.21 ± 5.33	13.71 ± 4.56	13.80 ± 4.19	0.085	0.990
Post‐treatment NIHSS scores	8.85 ± 3.77	9.90 ± 3.94	8.29 ± 3.87	0.555	0.010
Differences between pre‐ and post‐treatment NIHSS scores	6.35 ± 3.15	3.80 ± 2.13	5.51 ± 1.98	0.042	< 0.001
NIHSS scores at discharge	5.19 ± 2.84	8.28 ± 3.45	4.91 ± 2.27	0.769	< 0.001
Differences between pre‐treatment NIHSS scores and NIHSS scores at discharge	10.02 ± 3.75	5.42 ± 2.53	8.89 ± 3.02	0.029	< 0.001
NIHSS score reduction ratio	0.66 ± 0.12	0.40 ± 0.16	0.65 ± 0.12	0.857	< 0.001

*Note*: Continuous variables with normal distribution were expressed as mean ± standard deviation and compared between two groups with the independent sample *t*‐test. *p_a_* indicates comparison between the DMVOs‐MT and M1‐MT groups. *p_b_* indicates comparison between the DMVOs‐MT and DMVOs‐IVT groups. *p* < 0.05 indicates a significant difference.

**TABLE 5:** mRS scores and prognosis in the DMVOs‐MT, M1‐MT, and DMVOs‐IVT groups (*n* = 102).

Items	M1‐MT	DMVOs‐IVT	DMVOs‐MT	*p_a_*	*p_b_*
Pre‐treatment mRS scores	3.80 ± 0.81	3.93 ± 0.73	3.87 ± 0.71	0.521	0.562
mRS scores at discharge	3.15 ± 1.14	2.98 ± 1.00	2.65 ± 1.01	< 0.001	< 0.001
90‐day mRS scores	2.47 ± 2.11	2.90 ± 1.97	1.90 ± 2.00	0.050	< 0.001
Poor prognosis rate (90‐day mRS ≥ 3)	40 (39.22%)	54 (45.10%)	35 (34.31%)	0.468	0.007

*Note*: Continuous variables with normal distribution were expressed as mean ± standard deviation and compared between two groups with the independent sample *t*‐test. *p_a_* indicates comparison between the DMVOs‐MT and M1‐MT groups. *p_b_* indicates comparison between the DMVOs‐MT and DMVOs‐IVT groups. *p* < 0.05 indicates a significant difference.

### 3.4 Comparison of Adverse Event Incidence and Mortality in the DMVOs‐MT, M1‐MT, and DMVOs‐IVT Groups

The DMVOs‐MT, M1‐MT, and DMVOs‐IVT groups were insignificantly different in terms of the incidence of adverse events and mortality within 90 days post surgery, with comparable incidence rates of symptomatic intracranial and subarachnoid hemorrhages (all *p* > 0.05, Table 6). Overall, MT in patients with acute cerebral infarction due to DMVOs has a safety profile comparable to IVT in such patients and to MT in patients with acute cerebral infarction due to M1 segment occlusions.

**TABLE 6:** Adverse event incidence and mortality in the DMVOs‐MT, M1‐MT, and DMVOs‐IVT groups (*n* = 102).

Adverse events	M1‐MT	DMVOs‐IVT	DMVOs‐MT	*p_a_*	*p_b_*
Perforation	2 (1.96)	0 (0.00)	3 (2.94)	—	—
Dissection	3 (2.94)	0 (0.00)	2 (1.96)	—	—
Subarachnoid hemorrhage	2 (1.96)	4 (3.92)	3 (2.94)	—	—
Symptomatic intracranial hemorrhage	4 (3.92)	6 (5.88)	6 (5.88)	—	—
Adverse event incidence [*n* (%)]	11 (10.78)	10 (9.80)	14 (13.73)	0.522	0.385
Mortality within 90 days post surgery [*n* (%)]	15 (14.71)	11 (10.78)	14 (13.73)	0.841	0.522

*Note*: Categorical data were displayed as number of cases and percentages [*n* (%)] and compared between two groups with the chi‐square test. *p_a_* indicates comparison between the DMVOs‐MT and M1‐MT groups. *p_b_* indicates comparison between the DMVOs‐MT and DMVOs‐IVT group. *p* < 0.05 indicates a significant difference.

### 3.5 Analysis of Risk Factors for Poor Prognosis in Patients With Acute Cerebral Infarction due to DMVOs Following MT

According to the 90‐day mRS score (0–2 or 3–6 scores), patients with acute cerebral infarction due to DMVOs following MT were classified into a favorable prognosis group (90‐day mRS score ≤ 2, *n* = 67) and a poor prognosis group (90‐day mRS score ≥ 3, *n* = 35). With poor prognosis or not as the dependent variable, age, gender, smoking history, hypertension, diabetes, atrial fibrillation, hyperlipidemia, baseline ASPECTS, TOAST classification, time from symptom onset to puncture, time from puncture to recanalization, thrombectomy plan, number of thrombectomy, first‐pass recanalization, rescue plan, pre‐thrombectomy NIHSS scores, and pre‐treatment mRS scores were incorporated into the multivariate logistic regression analysis to determine the risk factors for poor prognosis in patients with acute cerebral infarction due to DMVOs following MT. The results manifested that diabetes (odds ratio [OR] = 22.226, 95% confidence interval [CI] = 2.525–195.621, *p* = 0.005), first‐pass recanalization (OR = 0.030, 95%CI = 0.003–0.282, *p* = 0.002), and pre‐thrombectomy NIHSS (OR = 1.335, 95%CI = 1.021–1.747, *p* = 0.035) were independent risk factors for dismal prognosis in patients with acute cerebral infarction due to DMVOs undergoing MT after adjustment for potential confounders (Table 7).

**TABLE 7:** Univariate and multivariate logistic regression analyses of risk factors for poor prognosis in patients with acute cerebral infarction due to DMVOs following MT.

Dependent variables	Univariate logistic regression analysis		Multivariate logistic regression analysis	
	OR (95%CI)	*P*	OR (95%CI)	*p*
Age	1.011 (0.967–1.058)	0.622	—	—
Gender	1.012 (0.440–2.331)	0.977	—	—
Smoking history	0.679 (0.286–1.612)	0.380	—	—
Hypertension	3.919 (1.556–9.871)	0.004	0.614 (0.072–5.226)	0.655
Diabetes	39.375 (11.296–137.248)	0.000	22.226 (2.525–195.621)	0.005
Atrial fibrillation	0.686 (0.297–1.585)	0.378	—	—
Hyperlipidemia	1.097 (0.484–2.487)	0.825	—	—
ASPECTS	1.100 (0.860–1.407)	0.447	—	—
TOAST classification	2.226 (0.954–5.193)	0.064	—	—
Time from symptom onset to puncture (m)	1.001 (0.998–1.004)	0.670	—	—
Time from puncture to recanalization (m)	1.003 (0.994–1.012)	0.541	—	—
Thrombectomy plan	0.919 (0.511–1.653)	0.779	—	—
Number of thrombectomy	9.798 (3.626–26.472)	0.000	5.982 (0.906–39.490)	0.063
First‐pass recanalization	0.021 (0.004–0.095)	0.000	0.030 (0.003–0.282)	0.002
Rescue plan	1.004 (0.416–2.422)	0.993	—	—
Pre‐thrombectomy NIHSS scores	1.531 (1.283–1.827)	0.000	1.335 (1.021–1.747)	0.035
Pre‐treatment mRS scores	1.668 (1.326–2.099)	0.000	1.302 (0.874–1.940)	0.195

## 4 Discussion

This study identified that MT may be superior to IVT regarding safety and efficacy in the treatment of patients with acute cerebral infarction due to DMVOs. Additionally, the efficacy and safety of MT in patients with acute cerebral infarction due to DMVOs were comparable to those in patients with acute cerebral infarction due to M1 segment occlusions. Such findings may provide a reference for the clinical application of MT in the treatment of cerebral infarction due to DMVOs.

Previous results unveiled that the efficacy of MT in the treatment of M2 segment occlusions was comparable to that in the treatment of M1 segment occlusions, as evidenced by no significant differences in the recanalization rate (Kniep et al. 2023; Saber et al. 2018), basically consistent with our research results. Importantly, patients with acute cerebral infarction due to large vessel occlusions exhibit a recanalization rate of 90% and a functional independence rate of approximately 50% at 90 days of MT treatment (Samaniego et al. 2018). In addition, a systematic review also supports that MT is an effective treatment option for patients with acute cerebral infarction due to DMVOs, evidenced by high successful recanalization and functional independence rates (Bilgin et al. 2023). Sarraj et al. reviewed five clinical trials and found that among 522 acute stroke patients with M2 segment occlusions, the prognosis of the MT group was superior to that of the IVT group, with no statistically significant differences in symptomatic intracranial hemorrhage between the two groups (Sarraj et al. 2016), highlighting that MT is necessary and effective in the treatment of M2 occlusions and cannot significantly increase surgery‐related complications. Concordantly, our findings also displayed that the recanalization rate of DMVOs‐MT was remarkably higher than that of DMVOs‐IVT, illustrating that MT outperforms IVT with thrombolysis in terms of efficacy in the treatment of patients with cerebral infarction due to DMVOs.

In the current study, no marked differences were found in the incidence of adverse events and mortality within 90 days post surgery among the DMVOs‐MT and M1‐MT groups; meanwhile, the DMVOs‐MT and M1‐MT groups exhibited comparable incidence rates of symptomatic intracranial and subarachnoid hemorrhages. Similarly, prior studies unveiled that after MT, the patients with M2 segment occlusions had no considerable differences in terms of mortality rates at 90 days and incidence of symptomatic intracerebral hemorrhage within 24 h compared with those with M1 segment occlusions (Liyis et al. 2023; Li et al. 2020; Xing et al. 2022; Wang et al. 2021). This study also displayed that after MT, compared with patients with M1 segment occlusions, patients with DMVOs had decreased differences between pre‐ and post‐treatment NIHSS scores and between pre‐treatment NIHSS scores and NIHSS scores at discharge, as well as no profound changes in the NIHSS score reduction ratio. In agreement with our results, a previous report corroborated no evident differences in the mean NIHSS scores both at admission and at discharge in patients with acute cerebral infarction due to M1 and M2 segment occlusions following MT (Dorn et al. 2015). Altogether, these results further demonstrate that MT is a safe and effective treatment for acute cerebral infarction due to both M1 segment occlusions and DMVOs.

Early neurological improvement is defined as a reduction of 8 or more points in NIHSS scores from the baseline score or NIHSS scores of 0 or 1 points at 24 h following MT, which is a strong independent predictor of favorable outcomes in patients with acute cerebral infarction due to DMVOs (Wang et al. 2022). Clinical outcomes can also be assessed according to mRS scores at day 90. Specifically, favorable outcomes are defined as 90‐day mRS scores ≤ 2, whereas unfavorable outcomes are defined as 90‐day mRS scores > 2 (Schregel et al. 2018). Subsequent results of our study disclosed that in patients with DMVOs, MT provoked better improvement in neurological function and lower poor prognosis rate than IVT, as shown by increased differences between pre‐ and post‐treatment NIHSS scores and between pre‐treatment NIHSS scores and NIHSS scores at discharge, as well as decreased 90‐day mRS scores. Compared with MT, IVT has the possibility of causing intracerebral hemorrhage and thrombus migration, delaying groin puncture, and increasing the expense of treatment; additionally, the recanalization rate following IVT varies from 10% to 30% based on the site of vessel occlusions and is particularly low in patients with high thrombus burden in the distal internal carotid artery (Xiong, Wakhloo, and Fisher 2022). In addition, a prior study included 171 patients with acute stroke (96 patients received EVT and 75 patients were given standard medications) for analysis. The results revealed that patients undergoing EVT showed better improvements in NIHSS scores at discharge and that in the distal M2 occlusion subgroup, EVT was prominently associated with higher rates of early neurological improvement and improved mRS scores at 3 months, with an incidence rate of symptomatic intracranial hemorrhage of 3.1% in the EVT group and 9.5% in the standard medication group (Marchal et al. 2022). Overall, these findings vindicate that for patients with DMVOs, MT has superior efficacy and safety to IVT.

According to logistic regression analysis results, diabetes, first‐pass recanalization, and pre‐thrombectomy NIHSS scores were independent risk factors for dismal prognosis in patients with acute cerebral infarction due to DMVOs after MT. In accordance with our results, a former study unveiled that stroke patients with diabetes had worse prognosis than patients without diabetes (Liu et al. 2021). More remarkably, another recently published research identified diabetes, mTICI scores, and NIHSS scores as risk factors associated with poor prognosis after endovascular therapy in patients with acute cerebral infarction (Huang et al. 2021). Prior multivariate analysis results demonstrated that diabetes and high NIHSS scores at admission were independent factors for poor 90‐day outcomes in patients undergoing endovascular therapy for acute cerebral infarction due to large vessel occlusions in acute anterior circulation (Zhang et al. 2021). Taken together, these studies indirectly support our findings that diabetes, first‐pass recanalization, and pre‐thrombectomy NIHSS scores may be independent risk factors for unfavorable prognosis in patients with acute cerebral infarction due to DMVOs after MT.

In conclusion, this study unravels the clinical efficacy and safety of MT in the treatment of acute cerebral infarction due to DMVOs. Specifically, the efficacy of MT in patients with acute cerebral infarction due to DMVOs is comparable to that of MT in patients with acute cerebral infarction due to M1 segment occlusions and is superior to that of IVT in patients with acute cerebral infarction due to DMVOs, with a favorable safety profile. Accordingly, MT is worth generalizing in the clinic. In addition, diabetes, first‐pass recanalization, and pre‐thrombectomy NIHSS scores were independent risk factors for dismal prognosis in patients with acute cerebral infarction due to DMVOs after MT. However, the current study had a small sample size. Therefore, studies with larger sample sizes are warranted to further investigate risk factors for the poor prognosis of patients with acute cerebral infarction due to DMVOs after MT.

## Author Contributions

**Lihong Zhang**: conceptualization, funding acquisition, visualization, validation, project administration, supervision. **Fanfan Su**: conceptualization, investigation, writing–review and editing, validation, project administration, supervision. **Jianhui Zhang**: investigation, funding acquisition, writing–review and editing, project administration, formal analysis, software. **Jia Xu**: investigation, writing–original draft, writing–review and editing, validation, resources, supervision, project administration. **Manhong Zhao**: funding acquisition, writing–original draft, methodology, formal analysis, software, data curation. **Di Li**: conceptualization, methodology, formal analysis, data curation, visualization. **Lin Yin**: writing–original draft, visualization, methodology, investigation, formal analysis, software, resources, data curation.

## Ethics Statement

The ethics committee of The Second Hospital of Dalian Medical University approved this study, which was performed in strict accordance with the *Declaration of Helsinki*.

## Consent

The authors have nothing to report.

## Conflicts of Interest

The authors declare no conflicts of interest.

### Peer Review

The peer review history for this article is available at https://publons.com/publon/10.1002/brb3.70119.

## Data Availability

The datasets used and analyzed during the current study are available from the corresponding author on reasonable request.
